# Incorporating efficient radial basis function networks and significant amino acid pairs for predicting GTP binding sites in transport proteins

**DOI:** 10.1186/s12859-016-1369-y

**Published:** 2016-12-22

**Authors:** Nguyen-Quoc-Khanh Le, Yu-Yen Ou

**Affiliations:** Department of Computer Science and Engineering, Yuan Ze University, Chung-Li, Taiwan

**Keywords:** Transport protein, GTP binding site, Position specific scoring matrix, Significant amino acid pairs, Radial basis function network

## Abstract

**Background:**

Guanonine-protein (G-protein) is known as molecular switches inside cells, and is very important in signals transmission from outside to inside cell. Especially in transport protein, most of G-proteins play an important role in membrane trafficking; necessary for transferring proteins and other molecules to a variety of destinations outside and inside of the cell. The function of membrane trafficking is controlled by G-proteins via Guanosine triphosphate (GTP) binding sites. The GTP binding sites active G-proteins initiated to membrane vesicles by interacting with specific effector proteins. Without the interaction from GTP binding sites, G-proteins could not be active in membrane trafficking and consequently cause many diseases, i.e., cancer, Parkinson… Thus it is very important to identify GTP binding sites in membrane trafficking, in particular, and in transport protein, in general.

**Results:**

We developed the proposed model with a cross-validation and examined with an independent dataset. We achieved an accuracy of 95.6% for evaluating with cross-validation and 98.7% for examining the performance with the independent data set. For newly discovered transport protein sequences, our approach performed remarkably better than similar methods such as GTPBinder, NsitePred and TargetSOS. Moreover, a friendly web server was developed for identifying GTP binding sites in transport proteins available for all users.

**Conclusions:**

We approached a computational technique using PSSM profiles and SAAPs for identifying GTP binding residues in transport proteins. When we included SAAPs into PSSM profiles, the predictive performance achieved a significant improvement in all measurement metrics. Furthermore, the proposed method could be a power tool for determining new proteins that belongs into GTP binding sites in transport proteins and can provide useful information for biologists.

## Background

Transport proteins are proteins interacted in cell membrane to bind and carry atoms and small molecules within cells and throughout the body. There are many different kinds of transport proteins, they are critical to the growth and life of all living organisms. Membrane trafficking is the important process in transport protein, in which proteins and other macromolecules are transferred to various destinations inside and outside of the cell. This process uses membrane-bound vesicles and vesicular transporters as mediates transport to establish the absorption of molecules within a vesicle.

**Fig. 1:**
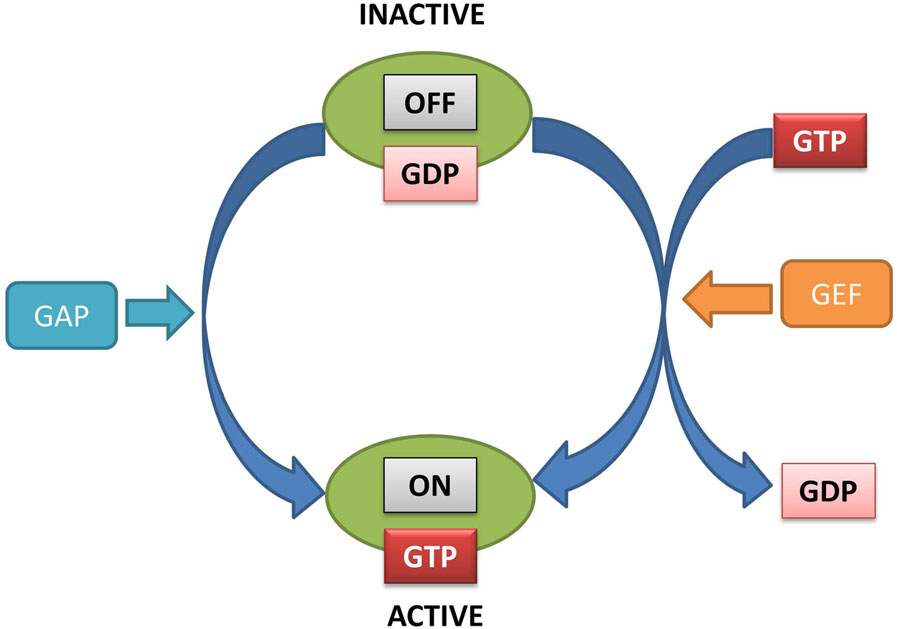
Process of GTP binding sites in transport proteins

To enforce membrane trafficking, G-proteins are activated to be recruited to membrane vesicles by interacting with specific effector proteins. Figure 1 indicates the process of G-protein in membrane trafficking. As shown in Fig. 1, G-protein operates as a molecular switch between GDP-bound inactive state and GTP-bound active state. These two states are controlled by guanine nucleotide exchange factors (GEFs) and GTPase activating proteins (GAPs). If G-protein binds GTP, it will be activated and involved in membrane trafficking. A number of studies determined that a functional loss of GTP binding sites in membrane trafficking has been implicated in a variety of human diseases (i.e., neurodegenerative, cancer, Parkinson [1–4] … So there is a need to develop techniques such as computational techniques for identifying GTP binding sites in membrane trafficking (especially in transport protein).

Because GTP binding sites have an important role in many biological processes, many people attempted to focus on them to perform research. A prominent study conducted on GTP binding sites is made by Chauhan [5]. They used support vector machines to predict GTP interacting residues. Hu [6] approached a new supervised over-sampling algorithm with application to protein-nucleotide binding residue prediction, including GTP binding sites. Chen [7] predicted and analysed of GTP binding residues using sequence and sequence-derived structural descriptors. In these studies, they also provided the free web servers for evaluating their methods. Susan and Peter [5] tried to analyse the role of GTP-binding proteins in transport along the exocytic pathway. Moreover, Yang and Rosenwald [3] summarized the functions of the monomeric GTP-binding proteins in macroautophagy in Saccharomyces cerevisiae. For the role of GTP binding sites in membrane trafficking, there are many researchers focusing on this field. One of them is from Hutagalung and Novick [1], they have reviewed the mechanisms of Rabs interacting with membrane trafficking. From this research, we understand the process of membrane trafficking and GTP binding sites in membrane trafficking.

Membrane and transport proteins are very important biological functions; thus many researchers have conducted their studies on this issue. For instance, Saier [6] built a web server containing many information of transport proteins from various living organisms. Next, Le [7] tried to developed a web server to predict FAD interacting residues in electron transport proteins with favourable results. Furthermore, Ren [8] developed transportDB, which is a complete database for predicting cellular membrane transporters. Chen [9] presented computational techniques to conduct prediction and analysis of transport proteins. After this work, the transport proteins are classified into four major classes with different transporter targets.

The present work developed machine learning techniques to identify GTP binding sites in transport proteins according to PSSM profiles and SAAPs. The cross-validation dataset is applied for developing the model and then we evaluation the model performance with independent data set. The accuracy from cross-validation and independent data set reached 95.6 and 98.7%, respectively. When we compared with the previous works presented by Chauhan [10], Hu [11] and Chen [12], the performance from proposed method improved significantly in all measure metrics. The proposed method could also predict the number of GTP binding sites with high accuracy and provide useful information for biologists. This study also provided a web server for presenting our method and can help biologists understand the function of GTP binding sites in transport proteins.

## Methods

**Fig. 2:**
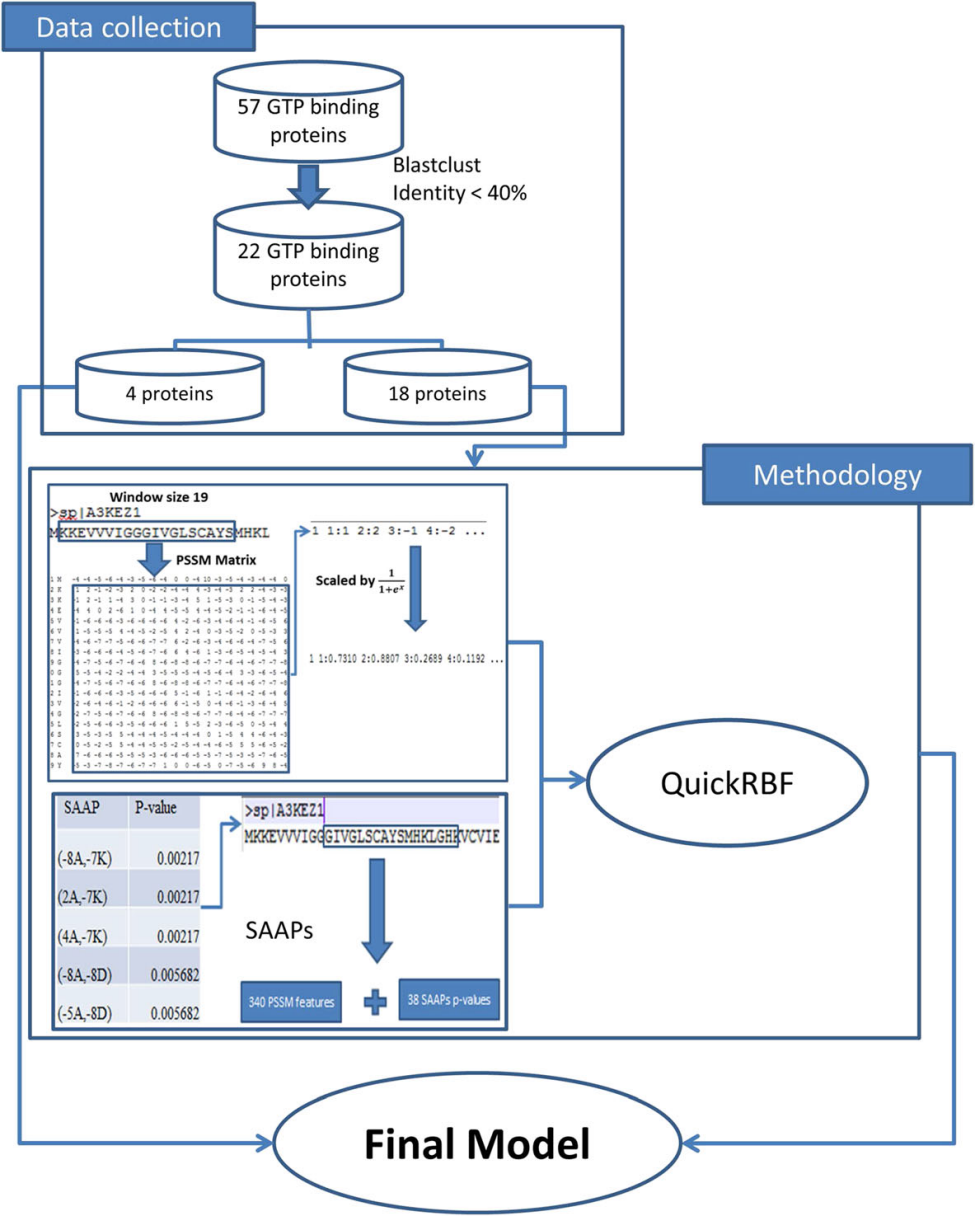
Whole architecture for predicting GTP binding sites in transport proteins

This study focused on predicting GTP binding sites in transport proteins. Figure 2 shows a whole architecture of the study, which contains three stages: data collection, feature set extraction, and model evaluation. According to this architecture, we presented a precise model using PSSM profiles and SAAPs for predicting GTP binding sites in transport proteins. We described the details of all processes as follows.

### Data collection

First of all, the data set about transport proteins is retrieved from the UniProt [13] database. In this collection step, we only selected sequences with the annotation “evidence at protein level” or “complete.” The detail query to retrieve transport proteins from UniProt is shown as follows:

*(annotation:(type:location AND membrane) AND existence:“evidence at protein level’ AND fragment:no AND reviewed:yes) AND (keyword: transport OR go: transport)*

After this step, 8772 transport proteins were collected. Next, we used the annotations from UniProt to collect GTP interacting residues in this data set. Note that in this step, we did not choose any GTP binding sites by similarity or by potential, we only choose GTP binding sites by experimental. After that we collected data on only 57 GTP binding proteins. To prevent overfitting in our model, we need to remove the similarity sequences inside the data set. We used BLAST [14] to perform this action, with sequence similarity of 40%. The number of transport proteins after remove redundant data is 22 proteins, and we used these 22 proteins as our final data set. We can see in Table 1, the 22 GTP binding proteins contain 364 GTP binding residues and 10434 non-GTP binding residues.

**Table 1:** All 22 GTP binding proteins using in the proposed study

Number of proteins	GTP binding sites	Non-GTP binding sites
22	364	10434

To build a model with high accuracy and avoid overfitting, we need to separate the data set into the cross-validation and independent data set. The proposed model will be fitted with the cross-validation data, and evaluated via the independent data set. The details of all data we used in this study are shown in Table 2. The number of training and testing dataset is chosen to have the balance positive data between each set. Finally, we used four GTP binding proteins in the transport protein (containing 52 GTP binding sites and 1710 non-GTP binding sites) as the independent data set. On the other hand, 18 GTP proteins (containing 312 GTP binding sites and 8774 non-GTP binding sites) contained in the cross-validation data set.

**Table 2:** The details of all 22 GTP binding proteins separated into independent dataset and cross-validation dataset

Independent dataset	Cross-validation dataset				
Q9UTE0	Q9ERI2	Q5S006	Q9H0F7	Q57986	P09527
Q8IXI2	Q9ULW5	Q41009	P33650	O75695	Q6IQ22
P60953	O35963	P93042	P42208	P51157	Q9UL25
P20606		Q9C0L9	A8INQ0	P62834	

### Sequence information

In many problems in predicting the secondary structure of proteins, sequence information is one of the first choice for researcher [15, 16]. This feature set used two dimension matrices with values represented 20 amino acid sequences. We computed all values of amino acids inside matrix and input them as a feature set. There are many types of matrix for performing sequence information. In this study, we applied three types of matrix, namely BINARY, PAM250 [16] and BLOSUM62 [17].

### Position specific scoring matrices profiles

PSSM is a common matrix in biology field to represent the sequences as motifs [18]. This matrix contains many score values represented for all amino acid in the original sequences. The row of PSSM shows the 20 amino acids and the column shows the original sequence of amino acids [19]. In several years, the PSSM has extensively been considered a trademark for representing the protein sequences. To identify protein sequences, the PSSM is proved better than the sequence information because it included values for full sequence at correct amino acid position. Many problems in bioinformatics, i.e., secondary protein structure used the PSSM and get the favourable results.

In this study, the PSSM profiles are generated from BLAST [14] and the non-redundant protein database. After this step, we retrieved the information from the PSSM profiles according to amino acids and their positions. The window size 19 also applied in this step to generate feature sets. Because the number of amino acid is 20, thus we have the matrix size 19 * 20 = 380 values. This matrix value should be converted into one vector and we extracted them for features. Finally we need to perform last step to scale data with the range from 0 to 1:

1 $$ \mathrm{F}\left(\mathrm{x}\right)=\frac{1}{1+ \exp \left(\hbox{-} x\right)} $$

### Significant amino acid pairs

**Fig. 3:**
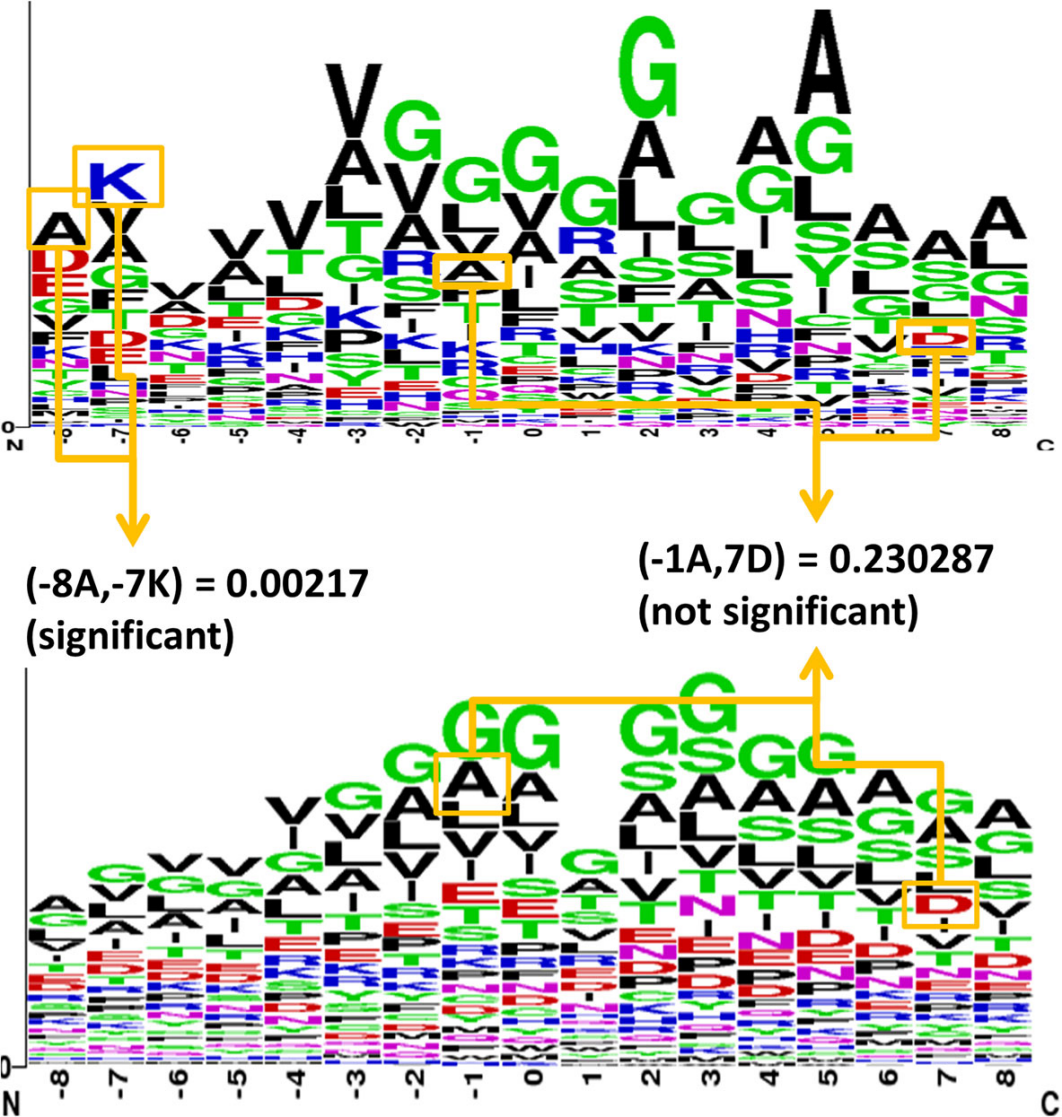
Methodology for identifying SAAP values in data set

To improve the predictive performance, we described SAAPs and combined with PSSM feature sets [7]. The SAAPs were generated from the cross-validation data set (22 proteins) to identify which pairs of amino acids appeared more frequency in this problem. To calculate the values for each amino acid pair surrounding the data set, we applied the formula:

2 $$ \mathrm{p}\hbox{-} \mathrm{valu}{\mathrm{e}}_k=\frac{\left(\underset{\mathrm{x}}{\mathrm{M}}\right)\left(\underset{\mathrm{n}\hbox{-} \mathrm{x}}{\mathrm{N}\hbox{-} \mathrm{M}}\right)}{\left(\underset{\mathrm{n}}{\mathrm{N}}\right)} $$

where N, M and (N-M) are the number of all proteins in the data sets, positive data sets, and negative data sets; n, x, and n-x are the number of sequences including a *k* ^th^ SAAP in the entire data set, positive data set, and negative data set. The detail method to compute all *p*-values from data sets is shown in Fig. 3.

We decided that each amino acid pair met significant level with *p*-value less than 0.030212. Thus there is much special information in these amino acid pairs and we could use them as an additional feature to identify GTP binding sites in transport proteins. To implement that, we added the selected SAAPs to the PSSM feature set in descending order and performed experiment. Finally, this study used 160 SAAPs as additional features combine to PSSM profiles for predicting GTP binding sites in transport proteins.

### Radial basis function networks

**Fig. 4:**
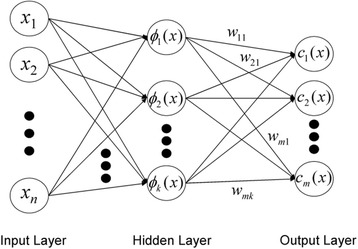
Architecture of the RBF network

For constructing RBF network, we developed the QuickRBF package [20] as a classifier. The architecture of RBF network is shown in Fig. 4. Moreover, we assigned a regular bandwidth of five for each kernel function is generated in the network. In this work, we selected the center data equal to the training data to get the best accuracy. Eventually, our classifier was used to discover GTP binding proteins in transport proteins to the output function value. We defined the details of the network structure and design in our previous article.[21].

In several bioinformatics and computational biology applications, RBF networks have been utilized in predicting cleavage sites in proteins [22], inter-residue contacts [23], and protein disorder [24]; moreover, they have been implemented for identifying β-barrel proteins [25], classifying transporters [26, 27], predicting O-linked glycosylation sites [28], FAD binding sites [7] and ubiquitin conjugation sites [29].

The output nodes in our RBF network determined with the expression as follows:

3 $$ {g}_j(x)={\displaystyle \sum_{i=1}^k{w}_{ji}\varphi \left(\left\Vert x-{\mu}_i\right\Vert; {\sigma}_i\right);} $$

where *g*_*j*_(*x*) denotes the function corresponding to the *j* ^th^ output node and is a linear combination of *k* radial basis functions $\varphi \left(\right)$ with center μ_*i*_ and bandwidth σ_*i*_ *.* Besides that, *w*_*ji*_ is the weight parameter for balancing data within the i^th^ hidden node and the j^th^ output node.

### Performance evaluation

Sensitivity, specificity, accuracy, and MCC (Matthew’s correlation coefficient) were used to evaluate the predictive performance. TP, FP, TN, FN are true positives, false positives, true negatives, and false negatives, respectively.

Sensitivity represents the percentage of GTP binding sites predicted correctly.

4 $$ \mathrm{Sensitivity}=\frac{\mathrm{TP}}{\mathrm{TP}+\mathrm{F}\mathrm{N}} $$

Specificity represents the percentage of non-GTP binding sites predicted correctly.

5 $$ \mathrm{Specificity}=\frac{\mathrm{TN}}{\mathrm{TN}+\mathrm{F}\mathrm{P}} $$

Accuracy represents the percentage of all GTP and non-GTP binding sites predicted correctly.

6 $$ \mathrm{Accuracy}=\frac{\mathrm{TP}+\mathrm{T}\mathrm{N}}{\mathrm{TP}+\mathrm{F}\mathrm{P}+\mathrm{T}\mathrm{N}+\mathrm{F}\mathrm{N}} $$

MCC represents the quality of prediction and prevent the unbalance data in model. A model prediction is perfect whenever the MCC value comes to 1.

7 $$ \mathrm{M}\mathrm{C}\mathrm{C}=\frac{\mathrm{TP}\times \mathrm{T}\mathrm{N}\hbox{-} \mathrm{F}\mathrm{P}\times \mathrm{F}\mathrm{N}}{\sqrt{\left(\mathrm{T}\mathrm{P}+\mathrm{F}\mathrm{P}\right)\left(\mathrm{T}\mathrm{P}+\mathrm{F}\mathrm{N}\right)\left(\mathrm{T}\mathrm{N}+\mathrm{F}\mathrm{P}\right)\left(\mathrm{T}\mathrm{N}+\mathrm{F}\mathrm{N}\right)}} $$

## Results and discussion

### Composition of amino acid analysis

**Fig. 5:**
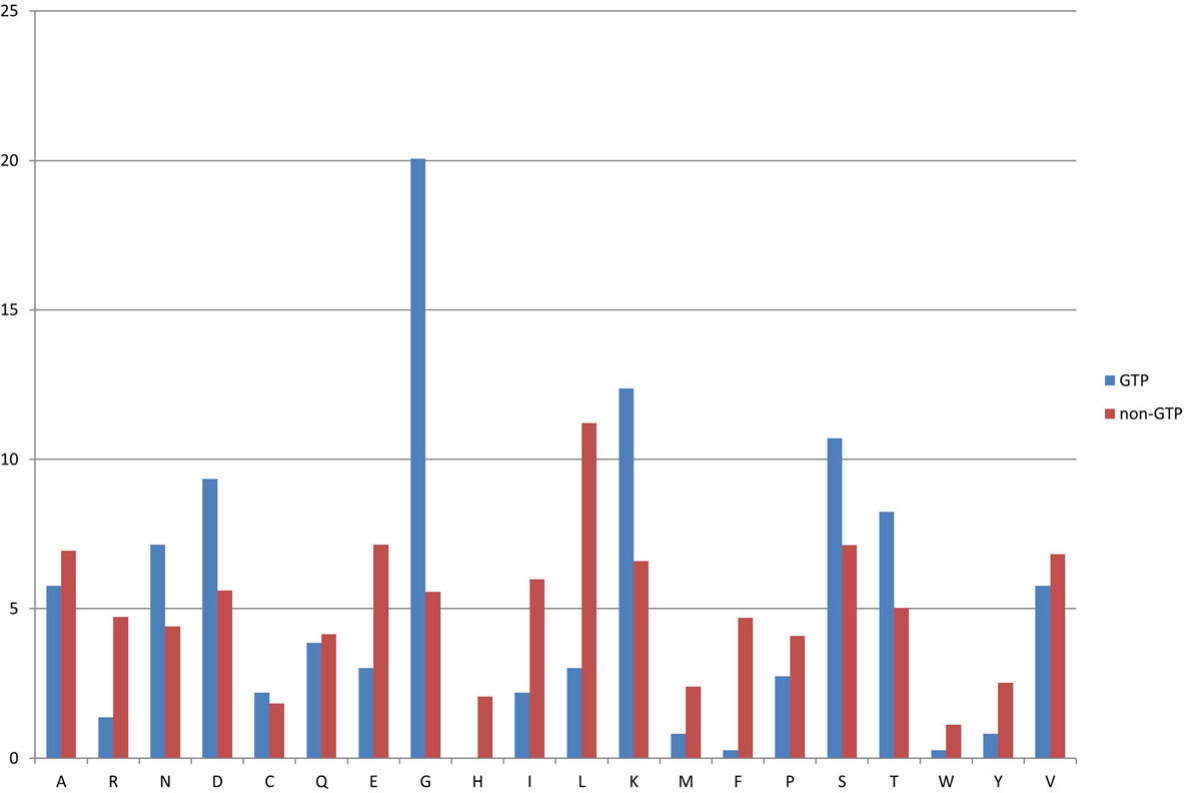
Composition of amino acid between GTP binding sites and non-GTP binding sites in data set

We calculated the occurrence frequency of all amino acids inside the dataset to analyse the composition of GTP binding sites and non-GTP binding sites in transport proteins. We can see the interaction in Fig. 5; highest occurrence frequency appeared with the amino acids G, K, S, and D. Therefore, these amino acids are the vital amino acids interacting with GTP binding sites in transport proteins. On the other hand, the amino acids L, S and D exceeded the low occurrence frequency in GTP binding sites in transport proteins.

### Comparison of the predictive performance with different window sizes

The proposed model is developed using the cross-validation dataset with 18 GTP binding proteins (including 312 GTP binding sites and 8774 non-GTP binding sites) in transport proteins. We selected the window sizes ranging from 13 to 19 for constructing our model. The measurement prediction executed with PSSM method and QuickRBF classifier. As shown in Table 3, the result did not improve too much when changing the window size. The better result was from window size 19, with the sensitivity, specificity, accuracy, and MCC were approximately 83.7%, 96%, 95.6%, and 0.58 respectively. Therefore we selected the performance result with a window size of 19 to develop our GTP binding model.

**Table 3:** Predicting GTP binding sites in the transport proteins with different window sizes

Window Size	True Positive	False Positive	True Negative	False Negative	Sens	Spec	Acc	MCC
WS13	259	334	8440	53	83	96.2	95.7	0.58
WS15	260	348	8426	52	83.3	96	95.6	0.58
WS17	249	409	8365	63	79.8	95.3	94.8	0.53
WS19	261	348	8426	51	83.7	96	95.6	0.58

**Fig. 6:**
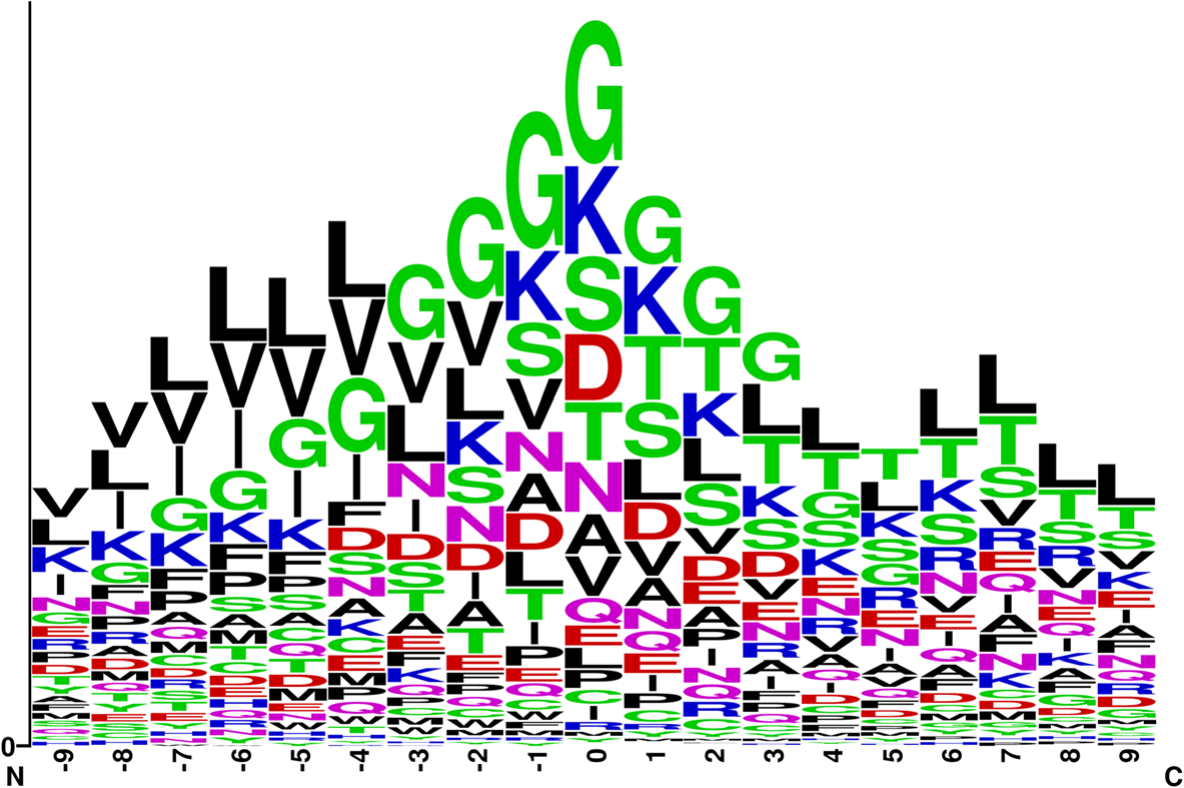
Sequence logo for 22 GTP binding proteins in transport proteins (generated from WebLogo)

Figure 6 plots the sequence frequency logo using WebLogo [30], which is a web application for sequence logos generator. We have cut-off the sequence with the window size 19 to have comparison between all fragments. This figure indicates that among all positions, there exist many amino acid differences from GTP binding sites in transport proteins. For instance, the amino acids G, K, S, and D contained some differences at positions 0. Therefore, we can identify GTP binding sites according to these amino acid differences.

### Comparison of the predictive performance with different feature sets

In this section, we performed the experiment for predicting GTP sites in transport proteins with different feature sets, including BINARY, BLOSUM62, PAM250, PSSM and SAAPs. We used both cross-validation and independent data set with window size 19 to execute prediction in this part. As shown in Table 4, the proposed method could perform better performance the other feature sets. We realized that the combination between SAAPs and PSSM profiles was favourable for developing the proposed work.

**Table 4:** Predicting GTP binding sites in the transport proteins with different feature sets

	Feature set	True Positive	False Positive	True Negative	False Negative	Sens	Spec	Acc	MCC
5-fold	BINARY	261	1951	6823	51	83.7	77.8	78	0.26
	BLOSUM62	232	412	8362	80	74.4	95.3	94.6	0.49
	PAM250	246	341	8433	66	78.8	96.1	95.5	0.56
	PSSM	260	351	8423	52	83.3	96	95.6	0.58
	PSSM + SAAPs	261	348	8426	51	83.7	96	95.6	0.58
Indept	BINARY	49	100	1610	3	94.2	94.2	94.2	0.54
	BLOSUM62	49	98	1612	3	94.2	94.3	94.3	0.54
	PAM250	49	71	1639	3	94.2	95.8	95.9	0.62
	PSSM	48	23	1687	4	92.3	98.7	98.5	0.78
	PSSM + SAAPs	49	20	1690	3	94.2	98.8	98.7	0.81

### ROC curve and AUC in predicting GTP binding sites in transport proteins

**Fig. 7:**
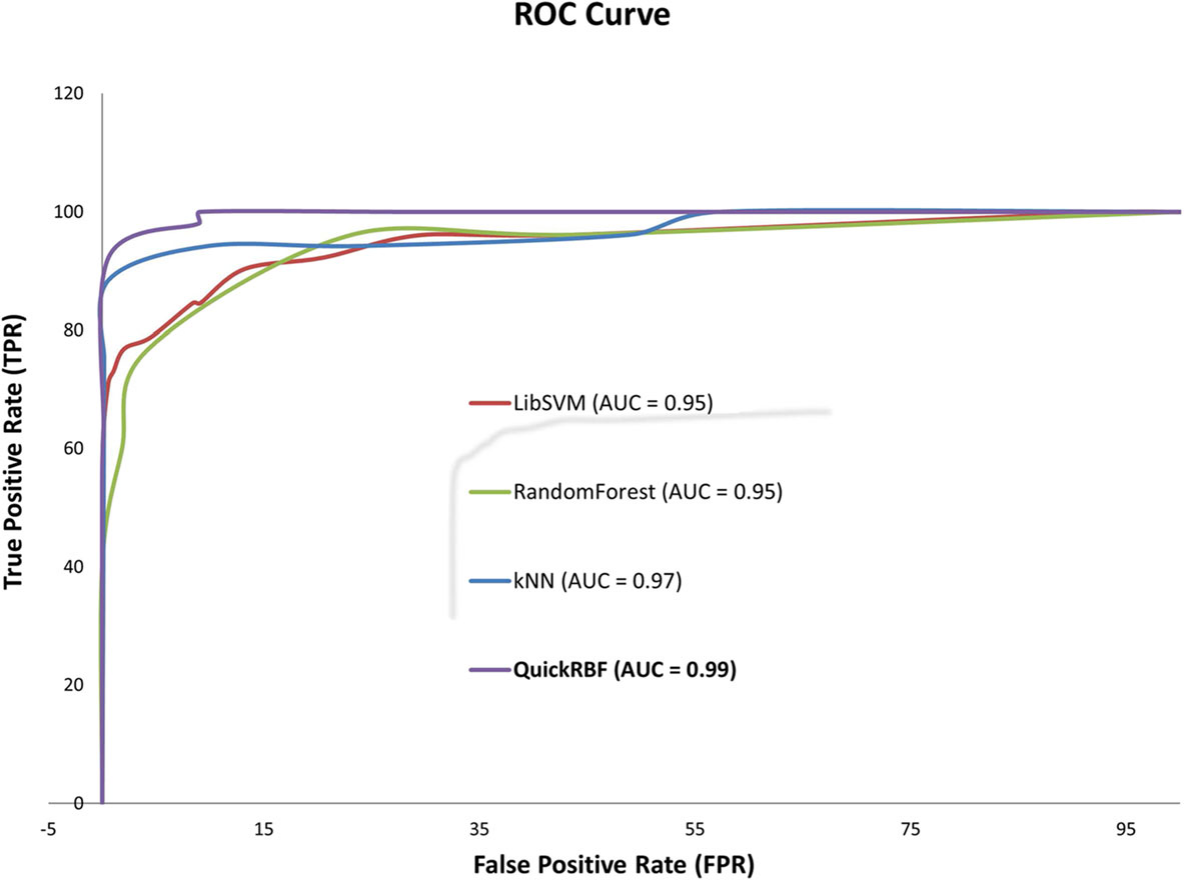
Comparison predictive performance between different classifiers with ROC Curve and AUC

Receiver operating characteristic (ROC) and area under the curve (AUC) are also applied as a significance analysis of the presented results [31]. The ROC curve plots from true positive rate and false positive rate based on our prediction results. In machine learning area, the ROC curve and AUC are the important metrics to present the accuracy of the test [32]. The AUC value is calculated from the ROC curve to represent the accuracy range. If the AUC comes to 1, we can detemine that our method perform accurately. In this study, our study reached higher AUC than other classifiers (AUC = 0.99), and therefore we could confirm that our classifier present better than others with this problem (Fig. 7).

### Comparison of predictive performance with different classifiers

In this section, some different classifiers are used in the cross-validation and independent data to have comparison with our method. There are many classifiers considered in this portion, i.e., kNN, RandomForest and LibSVM [33–35]. Table 5 shows the predictive performance from them, and our classifier performed well than the other classifiers. The sensitivity, specificity, accuracy, and MCC were respectively 83.7%, 96%, 95.6%, and 0.58 for cross-validation dataset. For independent dataset, the sensitivity, specificity, accuracy, and MCC were consequently 94.2%, 98.8%, 98.7%, and 0.81 Therefore we can use our classifier to present the proposed method to predict GTP binding sites in transport proteins.

**Table 5:** The comparison of predicting GTP binding sites in the transport proteins between different classifiers

	Feature set	True Positive	False Positive	True Negative	False Negative	Sens	Spec	Acc	MCC
5-fold	kNN	258	482	8287	54	82.7	94.5	94.1	0.51
	RandomForest	225	420	8349	87	72.1	95.2	94.4	0.48
	LibSVM	251	505	8264	61	80.4	94.2	93.8	0.49
	QuickRBF	261	348	8426	51	83.7	96	95.6	0.58
Indept	kNN	49	68	1641	3	94.2	96	96	0.61
	RandomForest	40	41	1668	12	76.9	97.6	97	0.6
	LibSVM	43	112	1597	9	82.7	93.4	93.1	0.45
	QuickRBF	49	20	1690	3	94.2	98.8	98.7	0.81

### Comparison of the proposed method with other published methods

We compared the predictive performance of our method with the previous studies from GTPBinder [10], NsitePred [12] and TargetSOS [11]. In the first comparison, we used the cross-validation and the independent dataset (including four transport proteins which contain 52 GTP binding sites and 1710 non-GTP binding sites) to perform the experiments with these methods. Table 6 shows that our proposed method performed remarkly better than the others in both cross-validation and independent data set.

**Table 6:** Predicting GTP binding sites in the transport proteins with other studies

	Cross-validation				Independent			
Feature set	Sens	Spec	Acc	MCC	Sens	Spec	Acc	MCC
Proposed method	83.7	96	95.6	0.58	94.2	98.8	98.7	0.81
GTPBinder	66.8	99.1	96.3	0.75	82.7	79.9	80	0.26
NsitePred	47.3	99.1	96.8	0.56	60.4	98.8	96.9	0.64
TargetSOS	47.3	99.5	97.4	0.6	61.9	98.8	97.1	0.66

Moreover, the second comparison is the predictive performance from two new discovered proteins after 2010, namely Q9H0F7 and A8INQ0. We applied our model in predicting these two proteins and compared the results with two studies GTPBinder [10] and TargetSOS approach [11]. The comparison performance in Table 7 indicatied that the proposed method improved better than the performance from GTPBinder method [10] and TargetSOS method [11].

**Table 7:** Predicting GTP binding sites in two newly discovered proteins

Classifier	True Positive	False Positive	True Negative	False Negative	Sens	Spec	Acc	MCC
Proposed Method								
Q9H0F7	15	9	162	0	100	99	99	0.84
A8INQ0	12	5	510	0	100	99	99	0.84
TargetSOS								
Q9H0F7	13	7	164	2	86.7	95.9	95.2	0.73
A8INQ0	10	14	501	2	83.3	97.3	97	0.58
GTPBinder								
Q9H0F7	11	33	138	4	73.3	80.7	80.1	0.35
A8INQ0	8	130	385	4	66.7	74.8	74.6	0.14

### Identification of new GTP binding sites in transport protein with the proposed method

We used our model in prediction of GTP binding sites in a set of human transport proteins, which retrieved from Swiss-Prot [36]. The BLAST also used in this section to remove redundant proteins with more than 30% similarity, and then remaining 100 proteins (including 21985 amino acids) were used to evaluate the model. After performing prediction with our approach, we found 938 GTP binding sites from this dataset. Therefore our model can be used to discover some new GTP binding sites in transport proteins with high accuracy.

### Web server for predicting GTP binding sites in transport protein

We developed the web server namely GTP-TP-RBF for representing our method in this study. GTP-TP-RBF was built from QuickRBF package to predict GTP binding sites in transport proteins according to PSSM profiles and SAAPs. The user can access our web server at http://140.138.155.226/~kahn/gtp-tp/. The web interface contains many friendly functions, in which users can understand the process and submit sequences easily. Moreover, we optimized the server performance to avoid the time consumption from submitting until getting results. Finally we tried to make a good display in the result page, thus users can retrieve the information easily. According to this web server, biologists can understand our presented work and discover new GTP binding sites in transport proteins.

## Conclusions

Because GTP binding sites have an important role in the process of transporters, predicting them is an important issue in bioinformatics and computational biology. This work presented an approach using radial basis function networks according to PSSM profiles and SAAPs for identifying GTP binding sites in transport proteins. We used the cross-validation to develop model and achieved the accuracy 98.7% when evaluating the performance with independent data set. Our predictive performance improved the accuracy by 18% and MCC by 0.55 when we compared with the general GTPBinder approach of Chauhan [10], Hu [11] and Chen [12]. Moreover, we have already provided a web server for presenting our method. Users can use our web server as an effective tool to understand the functions of GTP binding sites in transport proteins. They can identify some new GTP binding sites in transport proteins to serve their research. We expert that the contributions of this study will provide biologists many information for further research and enrich the bioinformatics field in future.

## Abbreviations

AUC: Area under curve
BLOSUM: Block substitution matrix
GAP: GTPase activating proteins
GDP: Guanosine diphosphate
GEF: Guanine nucleotide exchange factors
GTP: Guanosine triphosphate
MCC: Matthew’s correlation coefficient
PAM: Percent accepted mutation
PSSM: Position specific scoring matrix
RBF: Radial basis function
ROC: Receiver operating characteristic
SAAP: Significant amino acid pairs
